# Modular system for UV–vis-NIR radiation measurement with wireless communication

**DOI:** 10.1016/j.ohx.2021.e00236

**Published:** 2021-10-11

**Authors:** J.S. Botero-Valencia, M. Mejia-Herrera

**Affiliations:** Grupo de Sistemas de Control y Robótica, Instituto Tecnológico Metropolitano, Medellín, Colombia

**Keywords:** Spectrum measurement, Internet of things, Ultraviolet-UV, Near Infrared-NIR, Visible-VIS, Modular

## Abstract

Light wavelengths like Ultraviolet (UV) and Near-Infrared Radiation (NIR) are some topics of great interest for research in renewable energy, agriculture, architecture, interior design, and psychology. Due to the light spectrum influence in all these fields, it is necessary to develop instruments that facilitate their remote measurement and storage using Internet of Things technology. In this work, a modular system of sensors for UV–vis-NIR radiation measurement is presented. The system includes six multi-spectral sensors that allow 54 different measures. The acquired data can be sent to the cloud in real-time or stored in a micro SD memory in separate files (per sensor) to facilitate its reading, each data is time-stamped using Unix format, synchronized with a Network Time Protocol (NTP) server. The sensor enclosure was standardized in installation and all of them except the AS7265 have the same size, and were covered with a 1/8” PTFE sheet to take advantage of its diffuser characteristic. Finally, the sensors were mounted on a standard 2020 extruded aluminum guide rail, this rail allows to fix sensors in different distances and arrangements, bringing adaptability to the system.

## 1 Hardware in context

Lighting is variable of interest in different fields of study, such as renewable energy, indoor cultivation, chemistry, interior design, and even psychology [1], [2], [3]. Because light can be composed of radiation at different wavelengths, its quality can affect a system in various ways. For example, different UV radiation proportions in a lighting system could trigger health problems [4], [5], alter the energy production of solar panels [6], [7], cause vision problems [8], or cause early flowering or other modifications in some plant species [9], [10], [2]. That is why different commercial systems have been developed to study the composition of lighting systems or their behavior [11]. However, these systems are expensive or do not have real-time communication. These factors make it difficult development of research and integration into control systems to acquire the data automatically. Additionally, open hardware projects, in general, are a new way to increase knowledge democratization and expand the knowledge frontier faster. For this reason, it is necessary to support emerging initiatives where independent studies and research can be developed without the need for large and expensive laboratories. Additionally, one of the advantages of generating open hardware, such as the one developed in this work for the growth of research or data analysis, is the possibility of adding new measures or adjusting parameters that in most cases are complex tasks in instruments.

**Table:** 

**Specifications table:**	

**Hardware name**	Portable low-cost modular UV–vis-NIR acquisition device

**Subject area**	• Engineering
	• Instrumentation
	• Radiation measures
	• Internet of things

**Hardware type**	• Measuring physical properties and in-lab sensors
	• Field measurements and sensors
	• Electrical engineering and computer science

**Open source license**	Creative Commons Attribution-ShareAlike license

**Cost of hardware**	$229.25 USD

**Source file repository**	https://doi.org/10.17605/OSF.IO/J362K

Lighting studies usually focus on a specific wavelength, usually related to some research problem. Most cases use accurate and expensive equipment that sometimes tends to be complex for operation or some applications.Some articles present low-cost devices that facilitate the acquisition of lighting information for some wavelength [12], [13], [14], [15], [16]. Nevertheless, they are specific for some operation range(s). The following article presents the construction of a modular system for the acquisition of light information in UV-NIS and NIR bands with wireless communication for the transmission of the collected data, local storage on a micro SD memory, and a Li-Po battery for emergency operation. The designed system owns nine sensors that can be distributed to satisfy different requirements of light quality studies. However, due to their modular nature, it is possible to add more sensors, modify the position and orientation, as well as the wavelengths or range of interest. System sensor distribution consists of six multi-spectral sensors, two UV and two with NIR measurements. Therefore the system processes a total of 54 readings when all nine sensors operate together.Another essential factor in the development of light acquisition systems is the use of multi-spectral sensors. Such sensors permit the reconstruction of SPD from the available channels using artificial intelligence techniques [17] or measures to characterize light sources such as TM30-18 [18].

Lighting studies usually focus on a specific wavelength, usually related to some research problem. Most cases use accurate and expensive equipment that sometimes tends to be complex for operation or some applications. Some articles present low-cost devices that facilitate the acquisition of lighting information for some wavelengths [12], [13], [14], [15], [16]. Nevertheless, they are specific for some operation range(s). The following article presents the construction of a modular system for the acquisition of light information in UV-NIS and NIR bands with wireless communication for the transmission of the collected data, local storage on a micro SD memory, and a Li-Po battery for emergency operation. The designed system is suitable for indoor operation, and owns nine sensors that can be distributed to satisfy different requirements of light quality studies, reconstruct spectrum wavelength behavior, or estimate electric efficiency in a specific location by gathering 32 different bands. However, due to their modular nature, it is possible to add more sensors, modify the position and orientation, as well as the wavelengths or range of interest. Which brings adaptability to any specific study case without major modifications, reducing the number of needed devices when deploying diverse lighting research due to the IC bus. The system sensor distribution consists of six multi-spectral sensors, two UV and two with NIR measurements. Therefore the system processes a total of 54 readings when all nine sensors operate together. Another essential factor in the development of light acquisition systems is the use of multi-spectral sensors. Such sensors permit the reconstruction of SPD from the available channels using artificial intelligence techniques [17], measures to characterize light sources such as TM30-18 [18], evaluation of visual performance at some facilities [19], [20], the effect of some power distributions over ocular diseases [21], analysis in situ for some illumination systems [22], as well as other applications that might require the use of a spectrometer which is more expensive than the proposed system.

## 2 Hardware description

The system presented in this work is composed of different sensors to measure UV–vis-NIR radiation to facilitate the acquisition of such measurements or integrate it to tasks of automatic control of artificial sources. In total, the system owns up to nine sensors: four of them are multispectral, two principally UV, and three mainly in VIS. The multispectral sensors are the AS7262 with 6 channels in the VIS, the AS7263 with 6 channels in the NIR, the AS7265 with 3 photodetectors each with 6 channels, and the AS7341 with 11 channels in the VIS–NIR region. In the UV is the LTR390 and the VEML6075, these also provide compensation values in other regions of the spectrum. In the VIS, the VEML6030 is used, with an approximate response between 450–650 nm, and two TSL2591 are used, these have two channels, one between 500 and 950 nm and the other between 650 and 950 nm, the purpose of using two TSL2591 is to verify changes in distance, that is, although the system is modular, it is always proposed to use these two sensors and place them as far apart as possible to estimate a change in the region measured by the total array of sensors.

The use MCU is an ESP32 WROOM, selected due to its versatility, ease of acquisition in the market, and because it supports BT and WiFi communication. Considering that the I2C address of all multi-spectral sensors is the same and cannot be modified, it was necessary to use the TCA9548A multiplexer, as shown in 1, the multiplexer was also used to connect the two TSL2591 sensors, the sensors VEML6030, VEML6075, while LTR390 were connected directly to the I2C bus of the ESP32, using I2C OpenLog for data storage. It is important to mention that the design of system assembly does not need soldering, and all the sensors used have a Qwiic connector which facilitates their connection. The connector and the LiPo battery charging system is integrated into the development system selected in this project. Which makes it easier to use, increases usability and portability. Additionally, due to the SD card implementation, whenever the device lose internet connection, the data will still be gathered and saved into local storage for later study and comparison with the information sent via wifi to the cloud services provider (Ubidots in this case). This backup improves measurement storage security and facilitates research by integrating different data analysis tools provided by the cloud services. For the mechanical assembly, an enclosure was designed for each sensors, taking into account the field of view (FOV), and facilitating the access of the connectors (Qwiic). The enclosures of the sensors were covered with a 1/8 ”sheet of PTFE. Finally, using wedges, and M3 screws, the sensors can be assembled anywhere in a proposed structure of 2020 extruded aluminum guide rail. It is wanted to highlight, the idea that the sensors were in separate enclosures allows these sensors to be used more versatilely, the mounting style is just one example, for ease of installation, nevertheless, mounting can be adapted to different experimental conditions. One of the main advantages of this project is the diversity of measurements in the same system. Including 31 bands in specific wavelengths used to know characteristics of a particular source, reconstruct SPD, or determine measures that need or combine UV–vis-NIR, bringing the possibility of updating calculation methods to estimate novel illumination metrics proposed by the academic community. In the acquisition model, the possibility of storing in SD for a greater volume of data, or send it to the cloud, can support and facilitate research in multiple areas such as architecture, light engineering, and psychology, among others. The proposed architecture for data acquisition uses a blocking network which implies reading each sensor independently, the delay between measurements is about three seconds. And each measure is stored along with its respective timestamp to keep the variables chronology, facilitate the posterior analysis or use and ensure data collection. The system publishing frequency is a variable parameter in the code and can be adjusted for any desired application. Although the used sensors gather data down to the 0.25 Hz range in the default configuration, data is gathered and published with less than four seconds of difference, which is an advantage with respect to most of the commercial systems that do not store information or have an internet connection [24]. Additionally, as a security factor, when one of the sensors fails, an alert message indicates which one has to be replaced to keep tracking sensor correlation and therefore system accuracy. Table 1 shows the accuracy for each of the sensors used in this hardware, the experimental or theoretical type, refers to whether the accuracy was taken from the data-sheet (theoretical) or obtained by comparison (experimental), the number of measurements delivered by each sensor is also displayed. The proposed hardware provides several advantages:

• Supports the acquisition of UV–vis-NIR radiation for renewable energy, agriculture, architecture, interior design, and psychology purposes.
• Easy to integrate with smart systems to control artificial light sources, and thus improve the comfort conditions of the occupants.
• The system is modular and sensors can be removed to simplify acquisition and adapt them to a specific application.
• It can save the sensor data in an SD memory for saving energy for longer time data acquisition or when there is no Wi-Fi connection.

**Table 1:** Accuracy

Sensor	Type	Accuracy [%]	Unit	Measures
LTR390	Experimental	±24	counts	2
TSL2591	Experimental	±13	counts	2 x 2
VEML6075	Experimental	±15	counts	6
VEML6030	Experimental	±15	counts	1
AS7265	Theoretical	±12	counts	18
AS7263	Theoretical	±12	counts	6
AS7262	Theoretical	±12	counts	6
AS7341	Theoretical	±15	counts	11

## 3 Design files

**Fig. 1:**
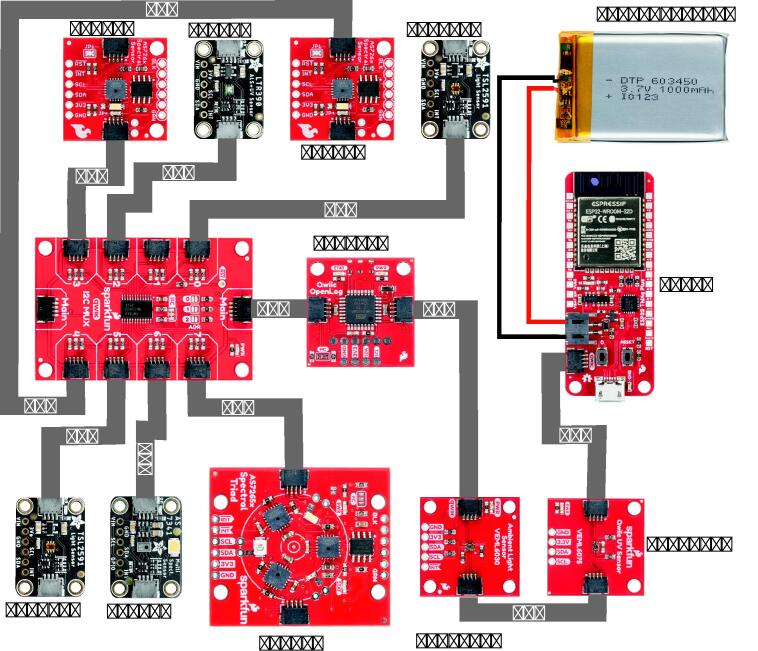
Schematic.

Fig. 1 shows the wiring between the electronic elements of the device. The main components are the ESP32 and the multiplexer TCA9548A. The sensors used are, AS7262, AS7263, AS7265 and AS7341 (multispectral), mainly UV, LTR390 and VEML6075, mainly VIS TSL2591 (2) and VEML6030. Additionally, the OpenLog is necessary to store the data in an SD memory and a LiPo battery to back up the power in case of failures, the charger is integrated into the ESP32 board.

### 3.1 Design files summary

**Table:** 

**Design file name**	**File type**	**Open source license**	**Location file**

0_Case_external	.stl	GNU GPL 3.0	https://osf.io/h9zwy/
0_Case_internal_Adafruit	.stl	GNU GPL 3.0	https://osf.io/by5ap/
0_Case_internal_Sparkfun	.stl	GNU GPL 3.0	https://osf.io/8p3he/
0_Case_external_AS7265	.stl	GNU GPL 3.0	https://osf.io/pd76k/
0_Case_internal_AS7265	.stl	GNU GPL 3.0	https://osf.io/r8keh/
0_Acrylic_Base_Generic	.stl	GNU GPL 3.0	https://osf.io/2d6tb/
1_Assemble	.png	GNU GPL 3.0	https://osf.io/n5h73/
1_Schematic	.eps	GNU GPL 3.0	https://osf.io/u8fet/
3_Code	.zip	GNU GPL 3.0	https://osf.io/qjf82/

• The *0*_*Acrylic*_*BaseGeneric* consists of a generic 3D printed grid plate for additional hardware if needed. Although the file is mainly designed for a 3D printer, this piece can be laser cut Table 2.

**Table 2:** Listed references and respective STL file for replication of the system.

**Reference**	**STL**	**Amount**
Acrylic base	0_Acrylic_BaseGeneric	1
Sensor Enclosure	0_Case_external	6
Adafruit Sensor holder	0_Case_internal_Adafruit	3
Sparkfun Sensor holder	0_Case_internal_Sparkfun	3
AS7265 Sensor Enclosure	0_Case_external_AS7265	1
AS7265 Sensor holder	0_Case_internal_AS7265	1
• The *0*_*Case*_*external* correspond to the external part of the enclosure for all the used sensors except the AS7265, and also serves as a frame designed for positioning a sheet of Teflon Table 2.
• The *0*_*Case*_*internal*_*Adafruit* is the internal part of the enclosure for the used Adafruit sensors (i.e. TSL2591, LTR390, AS7341, AS7262, AS7263), and complement the 0_Case_external Table 2.
• The *0*_*Case*_*internal*_*Sparkfun* is the internal part of the enclosure for the used Sparkfun sensors (i.e. VEML6030, VEML6030), and complement the 0_Case_external Table 2.
• The *0*_*Case*_*external*_*AS7265* correspond to the external part of the enclosure for the AS7265, and also serves as a frame designed for positioning a sheet of PTFE. Table 2
• The *0*_*Case*_*internal*_*AS7265* consists on the internal part of the enclosure for the AS7265, and complement the 0_Case_external Table 2.
• The *1*_*Assemble* consists in graphical aid to the system construction that contains the 3d assembled system.
• The *1*_*Schematic* for the connection of the electronic components.
• In the *3*_*Code.ino* file, is a C code to save into an SD memory, and post gathered information into the cloud.

## 4 Bill of materials

Table 3

**Table 3:** Bill of electronic components and suppliers web pages.

**Designator**	**Component**	**Qty**	**Unit cost**	**Total cost**	**Source of material**
**TSL2591**	Light sensor	2	$ 6,95	$ 13,90	https://t.ly/UKcD
**LTR390**	UV sensor	1	$ 4,95	$ 4,95	https://t.ly/ICpA
**AS7341**	10-channel	1	$ 15,95	$ 15,95	https://t.ly/qrm1
**AS7262**	VIS sensor	1	$ 25,95	$ 25,95	https://t.ly/CHRk
**AS7263**	NIR sensor	1	$ 25,95	$ 25,95	https://t.ly/6MhM
**AS7265X**	18-channel	1	$ 64,95	$ 64,95	https://t.ly/qUxr
**VEML6030**	Light sensor	1	$ 5,25	$ 5,25	https://t.ly/Un1V
**VEML6075**	UV sensor	1	$ 7,50	$ 7,50	https://t.ly/TgUy
**TCA9548A**	Mux	1	$ 11,95	$ 11,95	https://t.ly/DF4l
**Qwiic cable**	Cable	10	$ 1,50	$ 15,00	https://t.ly/wck7
**Qwiic OpenLo**g	SD log	1	$ 16,95	$ 16,95	https://t.ly/fvaa
**ESP32**	MCU	1	$ 20,95	$ 20,95	https://t.ly/1lhR
**Total**				$ 229,25	

## 5 Build instructions

The system was designed as a modular assembly that allows different sensor configurations and gives adaptability to many scenarios making it easier to develop lighting research. The structure is composed of three rectangular rail beams of size 20mm×20mm×200mm organized as in ‘H’ form. The beam unions consist of a couple of corner connection pieces and a trapezoidal nut juncture that allows fixing the beams and sensors placed on them as shown in Fig. 4b.

Beneath the ‘H’ structure, there is an acrylic sheet with a grid of perforations for M3 screws designed for the location of electronic components like batteries and micro-controllers. Nevertheless, due to the hole distribution, it is possible to add more elements if needed.

After structure construction sensor enclosure should be 3D printed, in this case, the task was done by using FDM (Fused deposition Material) technology. The printing process should be done using a proper set up to fit and align the models and reduce post processing job. The sensor cases consist of three components: (1) an internal part for fixing a sensor, (2) an external piece that protects the sensor, and allows positioning of a (3) sheet of Teflon that aids a homogeneous light distribution over the sensor. Each sensor case was designed to be placed whether on top, or at the laterals of the beams as shown in Fig. 4a, and each case STL file is available for 3D printing. To fix all the pieces it is necessary to use M3 bolts and nuts, some of them should be carefully inserted into the internal part of the cases. Although the external component is the same for every used sensor (except the AS7265), the inner piece varies according to each sensor geometry. It is highly recommendable to plug the sensors before closing the enclosure, to avoid cable damages or non desired stretches or bendings.

**Fig. 2:**
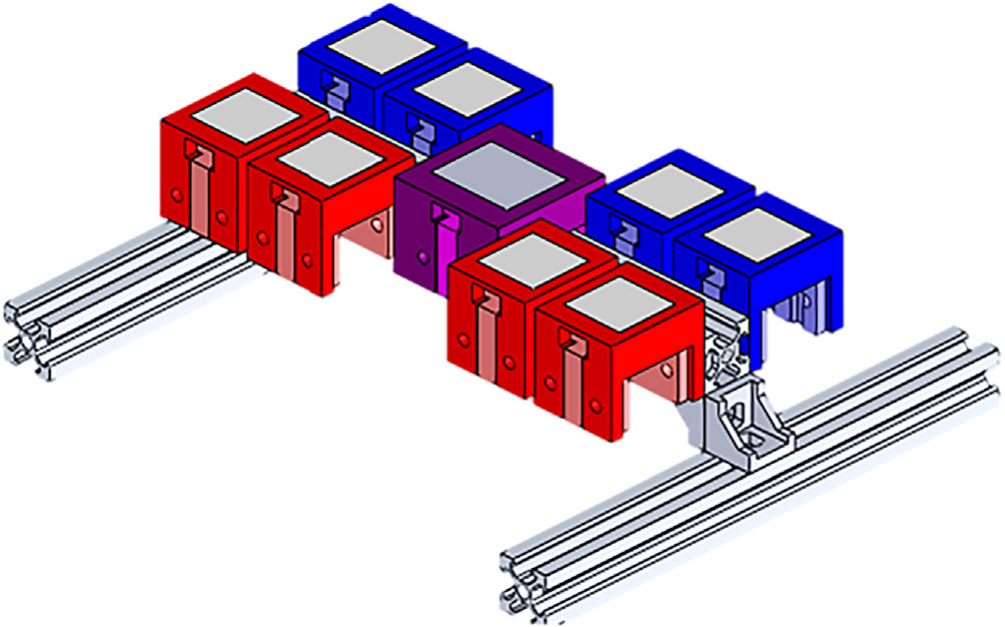
3D assembly and position of the components.

After the support structure assembly, and sensor enclosure constructions, the sensors should be placed in the ‘H’ structure by using trapezoidal nuts as mentioned before. Although different configurations can be done, we recommend symmetrical arrays that allows comparable illumination information, as shown in Fig. 4a. Finally, all the additional electronic components should be placed on the acrylic sheet and carefully plug together by following the Fig. 1, and the Fig. 2 shows the mechanical assembly of the proposed system.

## 6 Operation instructions

Operation of the developed system does not require any additional task than turn on the system and charge batteries occasionally. Nevertheless, before first time turning on, it does require adjusting some specific snippets of the available code to configure parameters such as Wifi ID, Wifi password, and the names for each sensor output log. This process can be done quickly by following the subsequent step-by-step information. Although the main code uses other files such as libraries and headers, these files should not be modified unless you need some specific hardware modification. Otherwise, and for the intended application, we will focus only on the Code.ino for the before-mentioned parameter configuration.

1. Download and install Arduino IDE, and set up LinKit One by following the instructions of the manufacturer.
2. Open Code.ino using Arduino IDE or any desired text editor
3. Modify Wifi network setup at lines 39 and 40 replacing, “Name” for your wifi ID and “Pass” for your private network password, see listing 1.
4. At line 41, 42, and 93 respectively. Replace “Token” with your web cloud provider token (Ubidots in this case), “Client” with your MQTT client name, and “industrial.api.ubidots.com” with your MQTT broker, see listing 1.
5. In the line 44, the user can modify the sampling period, that is, the interval in which the acquisition of all the connected sensors will be carried out, this time is given in milliseconds.
6. Lines 76 to 91 correspond to the output log names of the system. Such information can be printed in the system or saved into the predefined log files by setting “true” lines 95 and 96.
7. Once the code its adjusted, connect the MCU (Microcontroller Unity) via USB to the computer and press the upload button at the Arduino IDE, If the MCU terminal is checked, output messages are delivered showing errors or correct operation of the system.
8. After program uploading, the MCU will return multiple connection data, and the output is shown in Listing 2. Such information is included in the code to facilitate debugging for users. And it is the resulting sensor state of the scanning and enabling of the sensors connected to the I2C bus and the multiplexer. The OK status of each sensor indicates that it is connected and functional and ‘ER’ that it is disconnected or had a problem. The first two values (in brackets) represent the time in milliseconds of the MCU and the RTC (Real Time Clock), in Unix format.
9. Finally, in Listing 3, a typical example of running output is shown, it can be seen that: the MCU publishes the value of each variable, and their outputs are programmed within the code to facilitate debugging. Available gathered data is sent to the cloud for its processing and application, and the output is presented every 10 s in this case, nevertheless, this parameter can be adjusted in the code. The system acquires all sensors data within 2929 ms, this time can be affected by the type of used SD memory, however, each sensor is marked with its respective timestamp to know the acquisition instant.

It is important to consider which web service will be used to send the data to the cloud. In this case, the Ubidots company service was chosen. Since MQTT is used, if you want to change the operator, then you only have to update the code and libraries according to the selected service provider. Once the device is connected to a Wi-Fi or GPRS network and is properly located to acquire the data, it is only a matter to check the information on the web and using the data according to the application. Note, in case the user wants to make calculations directly in the MCU, the program code should be changed.

## 7 Validation and characterization

**Fig. 3:**
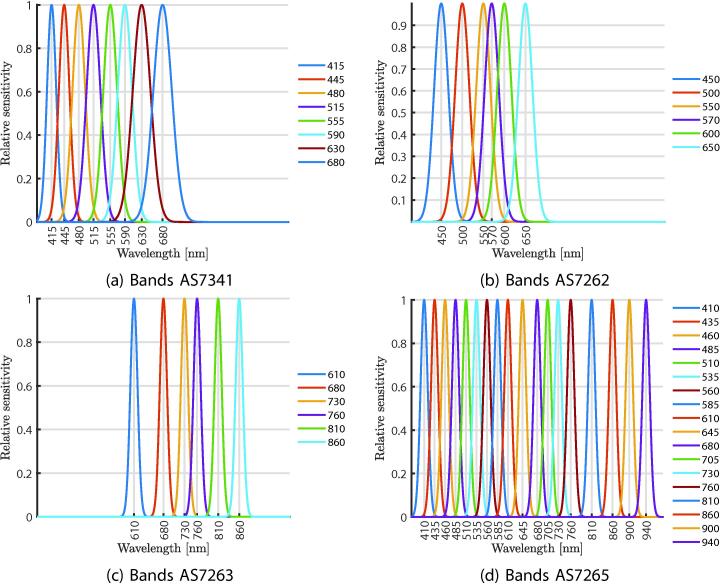
Bands available in multispectral sensors.

**Fig. 4:**
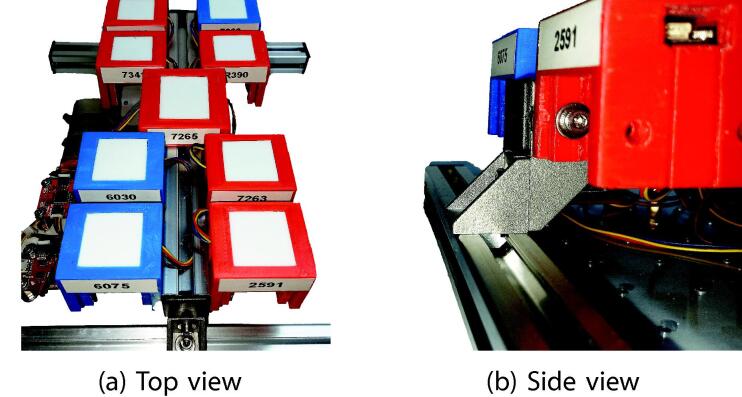
Photographs of the real device.

The Fig. 3 shows a summary of the available bands for each of the multi-spectral sensors. The system collects 38 bands where six are repeated bands, so there are 32 bands between 410 and 940 nm considering the central wavelength. In Fig. 4, photographs of proposed system assembly are shown.

**Fig. 5:**
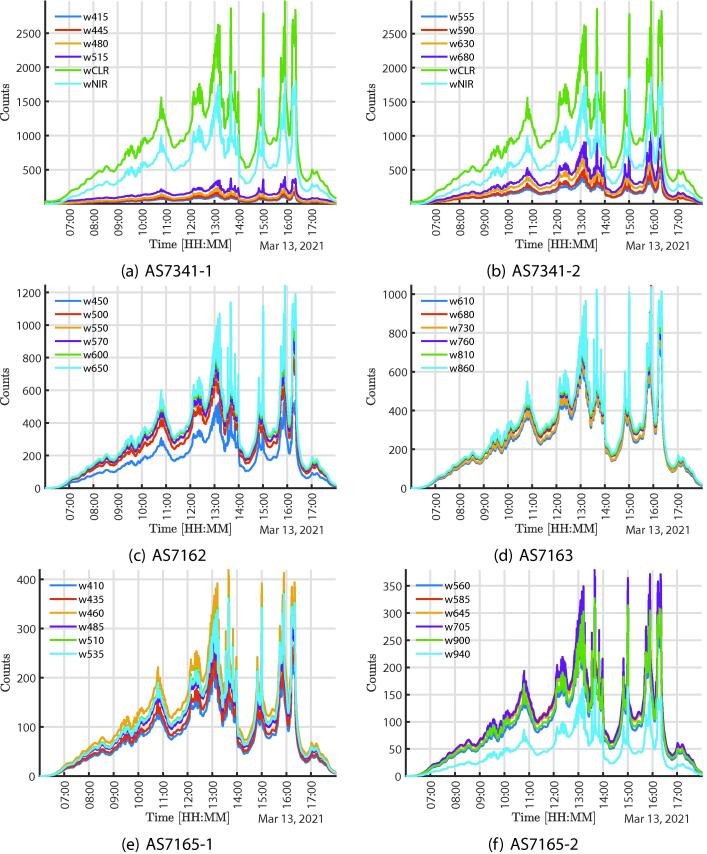
Data acquired from the Sun in a full day.

**Fig. 6:**
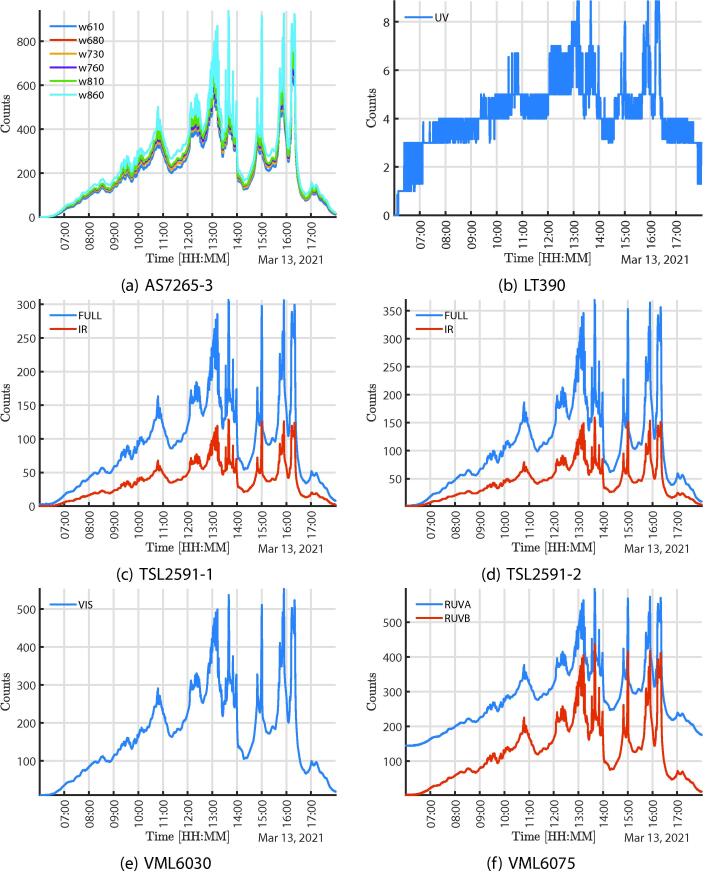
Data acquired from the Sun in a full day.

Fig. 5 shows the full-day solar acquisition response with the multispectral sensors locating the system behind a window facing the exterior. The graphs Fig. 5, Fig. 5, Fig. 5e–Fig. 5, Fig. 6a correspond to the sensors AS7341 and AS7265 information respectively, the AS7265 has three independent sensors, AS7265-1, AS7265-2, AS265-3, in the legends can see the specific wavelengths, data were divided to facilitate observation. It is important to notice that the sensitivity of the detectors can be adjusted digitally, which is an advantage to manage significant changes in the actual application. The purpose of this test is to perform an acquisition in an unstable environment since the behavior of solar radiation is affected by multiple environmental, atmospheric factors and the earth’s rotation.

Fig. 6 shows the full-day solar acquisition response mainly in the VIS and in the UV using the non-multi-spectral sensors locating the system behind a window facing the exterior. Fig. 6, Fig. 6, shows the response similarity of the TSL22 and TSL25 sensors, that correspond to the two TSL2591, which are recommended to be located far apart from each other in the assembly to use them as a reference and to bring uniformity to the measurements. The system also gathers the UV response, as shown in Fig. 6, Fig. 6, such sensors were added to the system mainly to monitor solar activity.

**Fig. 7:**
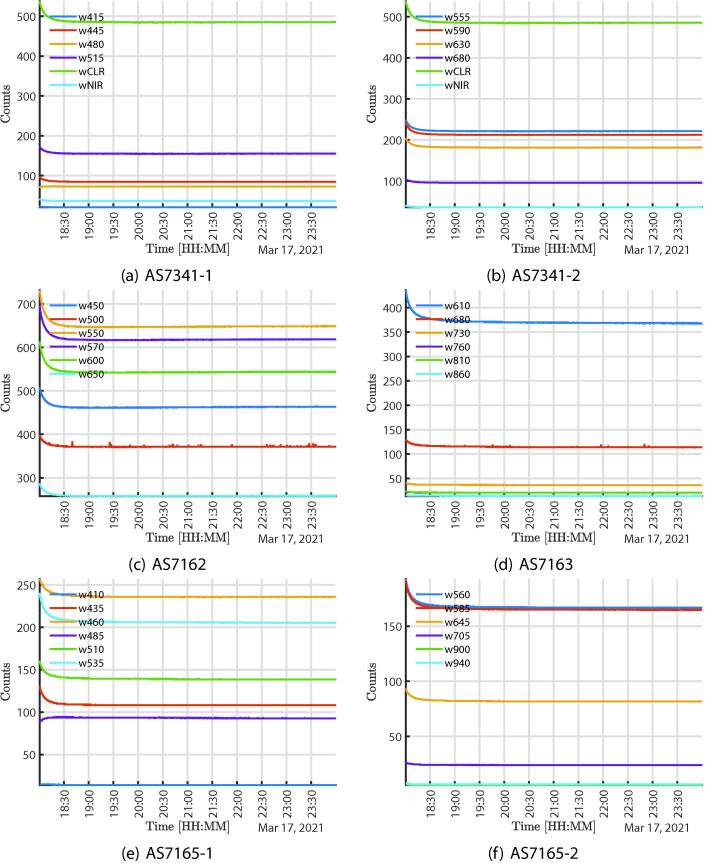
Data acquired from the artificial light source (LED) in 6 h.

Fig. 7 shows the response of multi-spectral sensors tested under a controlled environment, and illuminated with a 4000 K artificial LED light source for six hours. The purpose of this test was to show the stability of the measurements over time in a stable environment. For that reason, the system was illuminated by an artificial light source, in an environment without external influence from other sources.. The decrease in light output, which is consistent across all sensors at the beginning of the test, is a result of the time taken for the LED driver to stabilize the current. It can be seen that the infrared component is non-existent as expected and is presented as an application of the proposed system to estimate light efficiency.

**Fig. 8:**
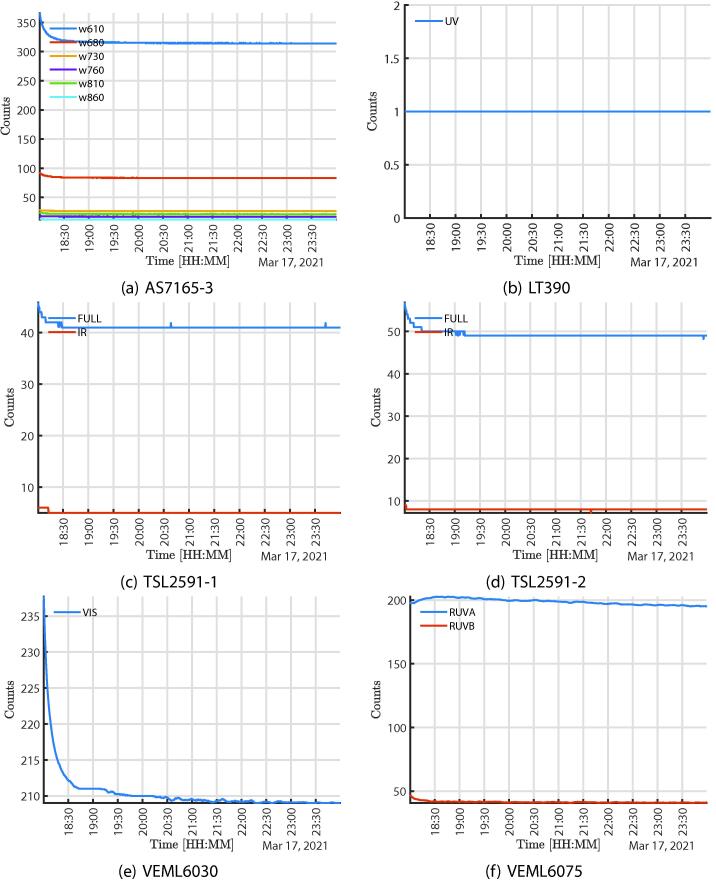
Data acquired from the artificial light source (LED) in 6 h.

Finally, in the Fig. 8, the response for the other sensors illuminated with the artificial light source LED is shown, this test was carried out for 6 h. Again it can be observed that the response in the NIR is non-existent and additionally that the LED source has a low level of UVA radiation and a very low level of UVB radiation. For indoor applications, one of the most important uses of this system would be light efficiency analysis and certifying light stability over time. This mainly because sometimes drivers, the electrical network, or obsolescence, generate degradation in the quality of the light artificial sources that should be considered.

The correlation matrix is used to establish the dependence between two variables, such dependence is a measure of the relationship between independent variables between −1 and 1. In Table 4, the correlation matrix for some selected channels is presented, the number of variables was reduced to simplify the presentation. The data correspond to the acquisition made of a full day of Sun, the high correlation is the result of the spectral composition of the Sun that is distributed in all the measured channels including the NIR and UV channels.

**Table 4:** Correlation matrix between channels Sun.

In contrast to the correlation matrix in the Table 4, in Table 5, the matrix for the six-hour acquisition of the artificial light source LED is presented. It can be seen that now the correlation with the UV and NIR channels is low, and the reason is the spectral composition of the LED sources that have relatively low values in these bands concerning the response in the VIS.

**Table 5:** Correlation matrix between channels, LED source.

Table 6 shows the normalized response on a scale from 0 to 100 of the cross-talk of the channels of the multispectral sensors used in this work. To obtain the results, a broad spectrum source EQ-99, monochromators and a spectrometer were used, the procedure can be seen in [23]. The FWHM of the monochromator is approximately 15 nm, it can be observed that there is a level of contamination between nearby channels (highlighted in yellow), however, the highest response was always given in the specific value of the channel (highlighted in green), that is It is important to clarify that the columns are the values in which the monochromator was set to make a measurement and the same values of the available bands were considered.

• Taking into account the number of sensors in the system, the battery should only be considered as a backup to a power failure and not to provide fully autonomy to the system as the battery capacity will not provide long-lasting power, and there is no solar or additional source power to keep the Lipo Battery charged. Instead, the ESP32 monitors the charging state and automatically shutdowns the charging stage to protect the battery when reaches the maximum capacity.
• The device is only suitable for indoor operation, weather conditions like rain, storms, or more aggressive environments can affect its operation.
• It is recommended to locate the system in areas with a low circulation of people because light reflections can affect the measurements.
• The system can store up to 8 GB of data in SD, the files are saved in separate files, one for each sensor and a system diagnostic file.
• The model presented in this work offers a large amount of information that can be useful in studies of architecture, light engineering, psychology, among others.
• With the acquired data, trend models can be developed over time to make predictions, to interpolate values with their mixture and to be able to predict some measurements in special applications, as is done with some commercial sensors in the case of UV.
• The system does not withstand the environmental conditions outside, it is not waterproof. However, it could be considered as future work, improving the enclosure of the sensors for an outdoor intended device.

**Table 6:** Crosstalk multispectral sensors.

## 8 Human and animal rights

No human or animal studies were conducted in this work.

## Declaration of Competing Interest

The authors declare that they have no known competing financial interests or personal relationships that could have appeared to influence the work reported in this paper.
